# *ZBTB16*-associated NK cell alterations reveal shared immunometabolic signatures linking primary Sjögren’s syndrome and type 1 diabetes mellitus

**DOI:** 10.3389/fimmu.2026.1871048

**Published:** 2026-08-18

**Authors:** Mingzhe Xin, Rui Mu, Yuxin Qian, Haonan Ding, Xuwen Wang, Zelong Hu, Shijia Huang, Ke Liu, Lei Jin

**Affiliations:** 1 Department of Stomatology, Nanjing Jinling Hospital, Affiliated Hospital of Medical School, Nanjing University, Nanjing, China; 2 Department of Orthodontics & Prosthodontics, The Second Affiliated Hospital, Zhejiang University School of Medicine, Hangzhou, China; 3 Department of Stomatology, The First Affiliated Hospital of Nanjing Medical University, Nanjing, China

**Keywords:** biomarker, immune-metabolic crosstalk, natural killer cells, primary Sjogren’s syndrome, type 1 diabetes mellitus, ZBTB16

## Abstract

**Background:**

Primary Sjögren’s syndrome (pSS) and type 1 diabetes mellitus (T1DM) share immune-inflammatory features, yet conserved pathogenic signatures linking these autoimmune disorders remain incompletely understood. The present research sought to uncover common molecular markers and dissect the underlying immune-metabolic cross-talk underlying pSS and T1DM.

**Methods:**

Gene expression profiles of patients with pSS and T1DM were retrieved from the Gene Expression Omnibus database, normalized, and corrected for batch effects prior to downstream analyses. Overlapping potential biomarkers were screened by integrating differential expression analysis, weighted gene co-expression network analysis and least absolute shrinkage and selection operator regression. Functional enrichment based on Gene Ontology and Kyoto Encyclopedia of Genes and Genomes databases was implemented to interpret gene biological properties, and a protein–protein interaction network was further established afterwards. Diagnostic performance was evaluated using receiver operating characteristic analysis. Experimental validation was conducted in non-obese diabetic (NOD) mice using quantitative PCR, immunohistochemistry, and flow cytometry. The CIBERSORT algorithm was adopted to quantify immune cell infiltration levels.

**Results:**

*ZBTB16* was identified as a shared hub biomarker in both pSS and T1DM and exhibited favorable diagnostic performance. Experimental validation confirmed significantly reduced *ZBTB16* expression in peripheral blood mononuclear cells, salivary gland tissues, and pancreatic tissues of NOD mice. Gene Set Enrichment Analysis indicated that *ZBTB16*-associated signatures were enriched in mitochondrial-related processes, neuroactive ligand-receptor interactions, and ribosome-related pathways. Immune infiltration analysis revealed that resting natural killer (NK) cells were positively correlated with *ZBTB16* expression in both diseases. Flow cytometric analysis further confirmed a reduced proportion of resting NK cells in peripheral blood of NOD mice, consistent with the CIBERSORT-based prediction.

**Conclusion:**

This study identifies *ZBTB16* as a shared biomarker linking pSS and T1DM. Reduced resting NK-cell abundance was consistently observed in both computational and experimental analyses, and bioinformatic correlation analysis suggested a positive association with *ZBTB16* expression. These findings provide evidence for shared molecular and immunological signatures underlying the two autoimmune disorders and support further investigation of the biological role and diagnostic value of *ZBTB16* in pSS and T1DM.

## 1 Introduction

Primary Sjögren’s syndrome (pSS) represents a chronic systemic autoimmune disorder that mainly impairs the function of exocrine glands, which in turn leads to enduring dry mouth and dry eye symptoms. Epidemiological investigations have demonstrated substantial variability in disease prevalence across populations, reflecting the influence of genetic background, environmental exposure, and diagnostic criteria (1). Beyond glandular involvement, pSS frequently presents with multi-organ manifestations affecting the respiratory, gastrointestinal, hepatic, and neurological systems, thereby contributing to considerable disease burden and reduced quality of life (2, 3). Accumulating evidence indicates that epithelial cells are not merely passive targets of immune-mediated injury in pSS, but instead actively participate in disease initiation and perpetuation (4–6). Aberrant activation of innate immune signaling pathways—including NF-κB, inflammasomes, and interferon-related cascades—has been documented in salivary gland epithelial cells (7). These alterations contribute to the recruitment and retention of CD4^+^ T cells and B cells within glandular tissues, leading to sustained immune–epithelial crosstalk (8). Consequently, epithelial-derived cytokines and chemokines amplify local inflammation, ultimately impairing salivary gland secretory function (9).

Type 1 diabetes mellitus (T1DM) is a prototypical autoimmune disorder driven by T-cell–mediated destruction of pancreatic β cells, resulting in absolute insulin deficiency and chronic hyperglycemia. In addition to metabolic dysregulation, T1DM is frequently accompanied by oral manifestations, including pronounced xerostomia. According to global epidemiological surveys, more than 9 million people globally suffer from T1DM (10). In T1DM, immune activation is characterized by the production of pro-inflammatory cytokines and activation of signaling pathways including NF-κB, which may indirectly affect salivary gland homeostasis via metabolic stress, microvascular damage, and neural dysfunction (11, 12).

Clinical observations and epidemiological studies have reported the coexistence of pSS and T1DM in a subset of patients, suggesting the presence of shared immunopathological mechanisms (13–16). However, xerostomia in individuals with T1DM is often attributed solely to hyperglycemia, potentially delaying recognition of underlying autoimmune glandular involvement. Although inflammatory mediators such as the IL-33/ST2 axis and IL-27 have been implicated in both conditions, the molecular basis underlying their concurrence remains poorly defined (17–19). Xerostomia symptoms in T1DM patients are often inaccurately ascribed to hyperglycemia alone, delaying the diagnosis and treatment of these concurrent conditions. However, the shared molecular mechanisms underlying pSS and T1DM remain poorly understood. Therefore, identifying shared biomarkers, dysregulated genes, and common molecular mechanisms through transcriptomic profiling is essential for elucidating the pathogenic links between these two autoimmune diseases.

**Figure 1:**
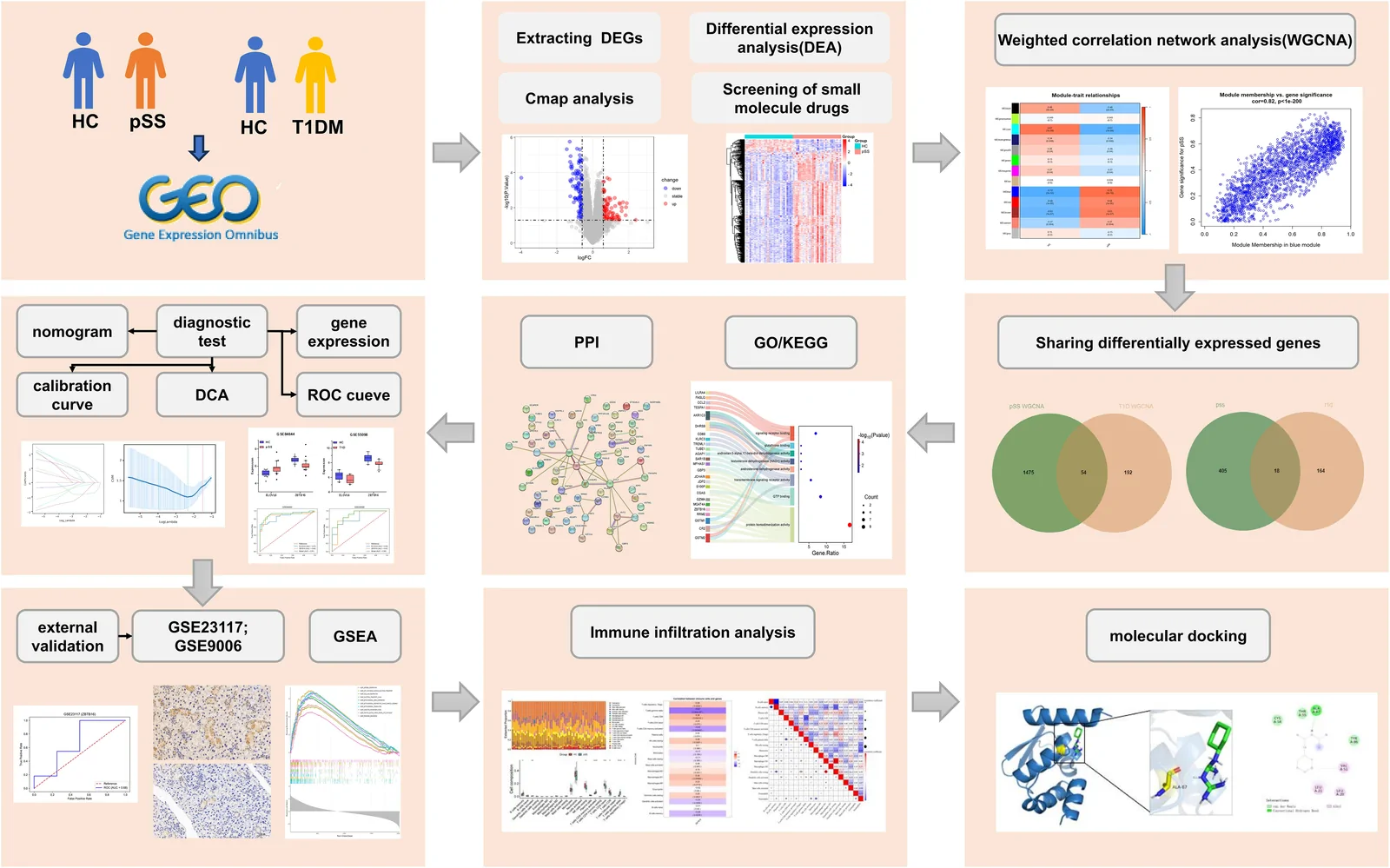
The flowchart of this study.

With the rapid development of transcriptomics and computational biology, integrative bioinformatics approaches now enable systematic exploration of disease-associated gene networks. In this work, we comprehensively mined publicly available gene expression datasets to identify shared molecular signatures between pSS and T1DM. By integrating differential expression analysis, weighted gene co-expression network analysis (WGCNA), machine-learning algorithms, immune infiltration profiling, and experimental validation, we aimed to identify shared biomarkers and characterize common immune-inflammatory and metabolic signatures underlying pSS and T1DM (**Figure 1**).

## 2 Methods

### 2.1 Data collection and preprocessing

Transcriptome datasets related to pSS and T1DM were retrieved from the GEO database, including GSE84844, GSE55098, GSE23117and GSE9006. GSE84844 comprises 30 pSS and 30 healthy control (HC) samples, while GSE55098 contains 12 T1DM and 10 HC samples. These two datasets served as the training cohorts. GSE23117 (11 pSS and 4 HC) and GSE9006 (39 T1DM and 20 HC) were used as independent validation cohorts. Available demographic characteristics extracted from the original GEO records are summarized in **Table 1**, with unreported information indicated accordingly.

**Table 1:** Detailed information on datasets.

Year	GSE number	Samples	Disease	Species	Attribute	Tissue source	Country of origin	Age (years)	Sex (F/M)
2017	GSE84844	30 patients and 30 controls	pSS	Homo sapiens	Training	Blood	Japan	24-76	59/1
2014	GSE55098	12 patients and 10 controls	T1DM	Homo sapiens	Training	Blood	China	Not reported	Not reported
2010	GSE23117	11 patients and 4 controls	pSS	Homo sapiens	Validation	minor salivary glands	USA	Not reported	Not reported
2007	GSE9006	39 patients and 20 controls	T1DM	Homo sapiens	Validation	Blood	USA	10-24	36/23

GSE9006 only selected 39 T1DM samples and 20 healthy samples for research.

Raw data were processed in R software (version 4.4.1), including data standardization, normalization, annotation, and removal of unannotated probes. When multiple probes mapped to the same gene symbol, the probe with the highest average expression value was selected. Batch effects among datasets were corrected using the “sva” package. The effectiveness of batch correction was evaluated by principal component analysis (PCA) before and after normalization.

### 2.2 Differential expression analysis (DEA)

The raw datasets were processed in R software. The “limma” package was utilized for normalization and to conduct DEA. To identify differentially expressed genes (DEGs), a significance threshold of P < 0.05 and |log2FC| > 0.585 was set between pSS and control samples, as well as T1DM and control groups. DEGs expression profiles were visualized using the “ggplot2” and “pheatmap” R packages, presented as volcano plots and hierarchical cluster heatmaps. Subsequently, DEGs were mapped onto the most clinically relevant gene modules for pSS, and Venn diagram analysis was employed to pinpoint intersecting key genes.

### 2.3 WGCNA analysis

We applied the hclust function to cluster the gene matrices, exclude outliers, and build a gene relationship network for the remaining sample data, selecting the optimal soft threshold to create a scale-free network model. The correlation among genes was calculated based on the chosen soft threshold and average connectivity, leading to the formation of an adjacency matrix. The topological overlap coefficient was transformed into a topological overlap matrix to aid module division. The module Eigengenes function was applied to calculate the eigenvalues of each module and to analyze their correlations with clinical phenotypes.

### 2.4 Functional enrichment analysis and protein–protein interaction (PPI) construction

Genes from clinically relevant modules were merged with the DEGs after removing duplicates. Subsequent GO and KEGG analyses, conducted via the Bioinformatics web platform, provided insights into the functional roles and signaling pathways of these genes.

For network construction, genes from the module most closely associated with clinical phenotypes were scored using the STRING database (https://cn.string-db.org/), applying a confidence threshold of 0.9 to identify crucial proteins. A PPI network diagram was then established, with the top 25 hub genes prioritized through the Maximal Clique Centrality (MCC) algorithm within the cytoHubba plugin of Cytoscape version 3.10.2.

### 2.5 Gene set enrichment analysis (GSEA)

GSEA was performed to explore the biological pathways associated with a single key biomarker gene. Specifically, for the selected diagnostic gene, Pearson correlation coefficients between its expression level and all other genes across samples were calculated. Based on these correlation coefficients, all genes were ranked in descending order to generate a pre-ranked gene list reflecting their degree of association with the target gene.

The ranked gene list was then submitted to the “clusterProfiler” R package, and enrichment analysis was conducted using the gseGO and gseKEGG functions to identify GO terms and KEGG pathways associated with the key biomarker gene.

### 2.6 Least absolute shrinkage and selection operator (LASSO) machine learning

LASSO regression analysis was implemented using the glmnet package in R. Model tuning was carried out by ten-fold cross-validation, and the value of λ that achieved the best performance was selected for subsequent analysis. Genes corresponding to λ.min were selected as candidate hub genes. Coefficient profiles and cross-validation curves were generated based on the glmnet framework. Result figures were generated using the online analysis platform (https://fast.statsape.com).

### 2.7 Characteristic genetic diagnostic tests and external validation

To evaluate the performance of the model, we used the “RMS” package to create a nomogram model and a calibration curve. The risk of pSS and T1DM was estimated using the total score produced from two genes, with predictive scores assigned to each gene in the nomogram. The net therapeutic benefit of the nomogram predictions was examined using decision curve analysis (DCA) with the “RMDA” program.

Expression levels of the identified characteristic genes were compared across training and validation cohorts (GSE84844, GSE55098, GSE23117, GSE9006). Receiver Operating Characteristic (ROC) curves were generated via the OECloud platform to evaluate the diagnostic utility of the genes and nomogram models. Additionally, gene expression distributions were visualized using GraphPad Prism 8 through box plot analysis.

### 2.8 Animal

All animals were sourced from GemPharmatech Co., Ltd. (Nanjing, China). NOD/LtJ mice (n =8) were purchased as the experimental model, while healthy Institute of Cancer Research (ICR) mice (n = 8) served as the controls. Mice were maintained under standardized conditions, with a regulated temperature of 24 ± 2 °C, relative humidity of 55 ± 10%, and a 12-hour light/dark cycle. Peripheral blood samples were collected via ocular extraction, and submandibular glands were harvested following euthanasia by cervical dislocation. All animal procedures were approved by the Experimental Animal Ethics Committee of Nanjing Medical University (Approval ID: IACUC-2503071).

### 2.9 Immunohistochemical analysis

As soon as they were removed, the submandibular glands were immersed in 4% paraformaldehyde (P0099, Beyotime) for at least one day. Before embedding, tissues were dehydrated, cleared, and infiltrated with paraffin after fixation. Using a microtome (RM2016, Leica), 3 μm thick histological slices were generated. Sections were rehydrated using a graded ethanol series after being dewaxed in xylene. Endogenous peroxidase activity was inhibited using a 3% hydrogen (H112520, Aladdin) peroxide solution, and target epitopes were exposed using antigen retrieval. Applying 3% BSA (A8020, Solarbio) within an immunohistochemical barrier and incubating it for 30 minutes blocked nonspecific binding. After the primary antibodies were incubated for one to two hours or overnight at 4 °C, the secondary antibodies were added. Subsequently, PBS was used to extensively rinse off any unbound antibodies. 3,3’-Diaminobenzidine (HY-W014212, MCE) was used for chromogenic development and hematoxylin for nuclear counterstaining to produce visualization. After that, the sections were dehydrated using an ethanol gradient, rinsed in xylene (A530011, Sangon Biotech), and finally mounted with coverslips. Bright-field microscopy (DM3000, Leica) was employed for examination. Antibodies used included Anti-*ZBTB16* (HPA001499, Sigma-Aldrich).

### 2.10 Quantitative real-time polymerase chain reaction

After TRIzol (15596026CN, Thermo Fisher) mediated total RNA extraction from mouse peripheral blood mononuclear cells (PBMCs), a reverse transcription reaction (R333-01, Vazyme) was used to transform the recovered RNA into cDNA. The reaction wells were filled with the prepared and mixed reaction system according to the specific needs. The 40-cycle PCR amplification process included denaturing the DNA at 95 °C for 30 s, denaturing it again for 15 s, and then annealing or extending it at 60 °C for 30 s. All during the amplification process, signals of fluorescence were recorded. We used the GAPDH internal control to standardize the target gene expression levels. The primer sequence is provided in the attachment (**Supplementary Table 2**).

### 2.11 Immune cell infiltration (ICI) analysis

To evaluate the relative proportions of immune cell subsets in the GSE84844, GSE55098 datasets, the CIBERSORT method was used with samples. The dissimilarities in the distributions of immune cells were evaluated with the help of the Wilcoxon rank-sum test. Heatmaps were generated through bioinformatics platforms (https://www.bioincloud.tech/) to facilitate data analysis and visualization.

### 2.12 Flow cytometry analysis

Single-cell suspensions were obtained from peripheral blood collected from NOD and control mice. Following red blood cell lysis, leukocytes were resuspended in FACS buffer and incubated with fluorochrome-conjugated antibodies against CD45 (147708, BioLegend), NKp46 (137603, BioLegend), and CD69 (104523, BioLegend) for 30 min at 4 °C in the dark. Debris and doublets were excluded before analysis using FSC-A/FSC-H gating. Data acquisition was carried out on a BD FACSymphony™ A1 flow cytometer and subsequently analyzed with FlowJo software (version 10). The gating strategy for NK-cell analysis is provided in **Supplementary Figure S5**.

### 2.13 Small molecule drug screening and molecular docking

Potential therapeutic compounds were identified using the Connectivity Map (CMap) database based on differentially expressed gene signatures from pSS and T1DM. Top upregulated and downregulated genes were submitted for analysis to identify compounds with potential reversal effects. Protein structures were obtained from AlphaFold, and ligand structures were retrieved from PubChem. Protein structures were first prepared by removing water molecules and adding hydrogen atoms. Docking analysis was carried out with AutoDock Vina (v1.2.0). The resulting binding conformations were further examined using PyMOL (v3.1.0) and Discovery Studio Visualizer (2019). Molecular dynamics simulations were subsequently performed in GROMACS (v2022), with force-field topology files generated through the pdb2gmx module.

## 3 Results

### 3.1 Identification of shared transcriptomic signatures in pSS and T1DM

**Figure 2:**
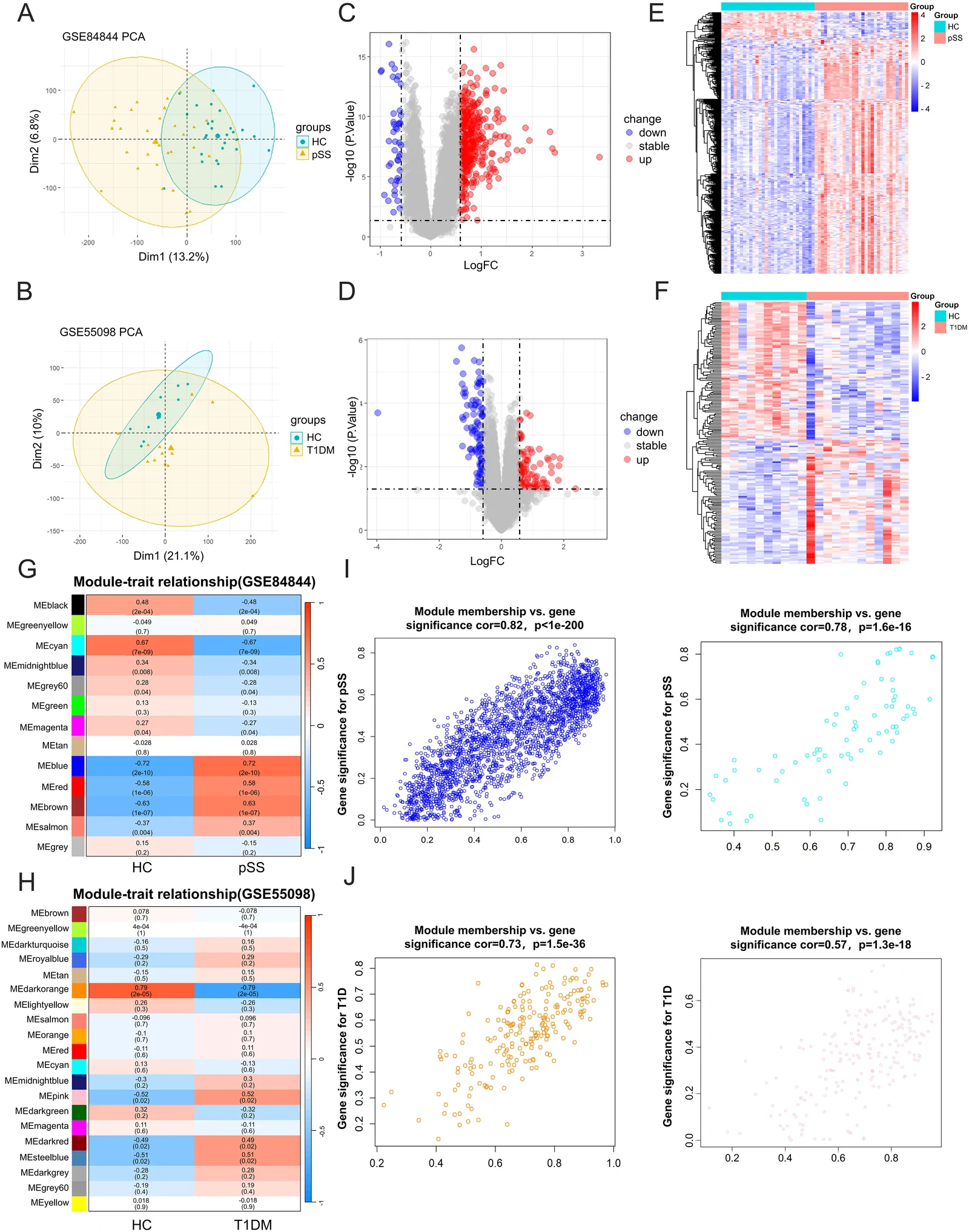
Identification of disease-specific transcriptomic alterations in pSS and T1DM. **(A)** Principal component analysis (PCA) plots of pSS and HC. **(B)** Principal component analysis (PCA) plots of T1DM and HC. **(C, D)** Volcano plots showing differentially expressed genes, red indicates upregulated genes and blue indicates downregulated genes. **(E, F)** Heatmaps showing hierarchical clustering of differentially expressed genes in pSS, T1DM. **(G, H)** Module–trait relationship heatmaps, red represents positive correlation, blue represents negative correlation. **(I, J)** Scatter plots showing the relationship between module membership and gene significance. pSS, primary Sjögren’s syndrome; T1DM, type 1 diabetes mellitus; HC, healthy control.

To screen key genes for PSS and T1DM, differential expression analyses were performed on datasets GSE84844 and GSE55098, respectively. PCA plots demonstrated clear separation between pSS and healthy control samples in GSE84844 (**Figure 2A**) and between T1DM and healthy control samples in GSE55098 (**Figure 2B**). Differential expression analysis identified 423 DEGs in pSS (379 upregulated and 44 downregulated) and 182 DEGs in T1DM (86 upregulated and 96 downregulated) (**Figures 2C–F**).

WGCNA was performed on the GSE84844 and GSE55098 datasets to construct co-expression modules correlated with pSS and T1DM. Two outlier samples in GSE84844 and one outlier sample in GSE55098 were identified and removed. For both datasets, an optimal soft threshold of 6 (with pSS R^2^ = 0.84, T1DM R^2^ = 0.88) was selected to construct a scale-free network. After merging modules containing highly correlated characteristic genes, 12 gene modules for GSE84844 and 20 for GSE55098 were identified. The module-trait relationships were assessed and visualized, revealing that key disease-associated modules, including the blue and cyan modules in pSS (1,597 genes) and the dark orange and pink modules in T1DM (246 genes) (**Figures 2G–J**) (**Supplementary Figures S1, S2**).

**Figure 3:**
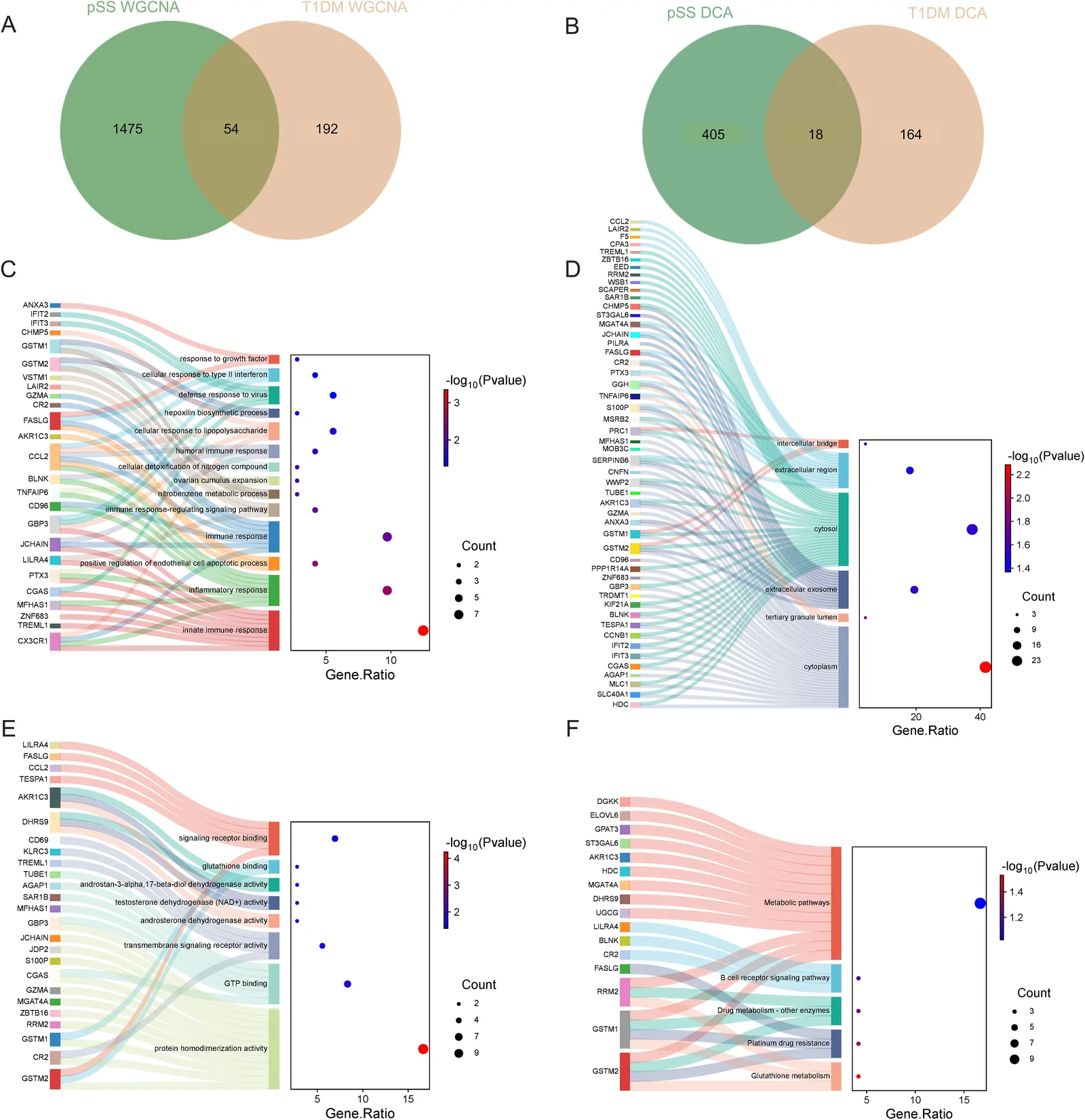
Identification of shared genes and functional enrichment in pSS and T1DM. **(A, B)** Venn diagrams showing the identification of shared genes. **(C–F)** GO and KEGG enrichment analysis results. pSS, primary Sjögren’s syndrome; T1DM, type 1 diabetes mellitus; GO, Gene Ontology; KEGG, Kyoto Encyclopedia of Genes and Genomes.

To identify genes potentially shared between the two diseases, differentially expressed genes (DEGs) were overlapped with genes from key WGCNA modules, resulting in 72 common genes in both pSS and T1DM (**Figures 3A, B**). Subsequent functional enrichment analysis indicated that these overlapping genes were predominantly associated with innate immune responses, regulation of inflammatory processes, and glutathione metabolism (**Figures 3C–F**).

### 3.2 *ZBTB16* was identified as a shared hub biomarker in pSS and T1DM

**Figure 4:**
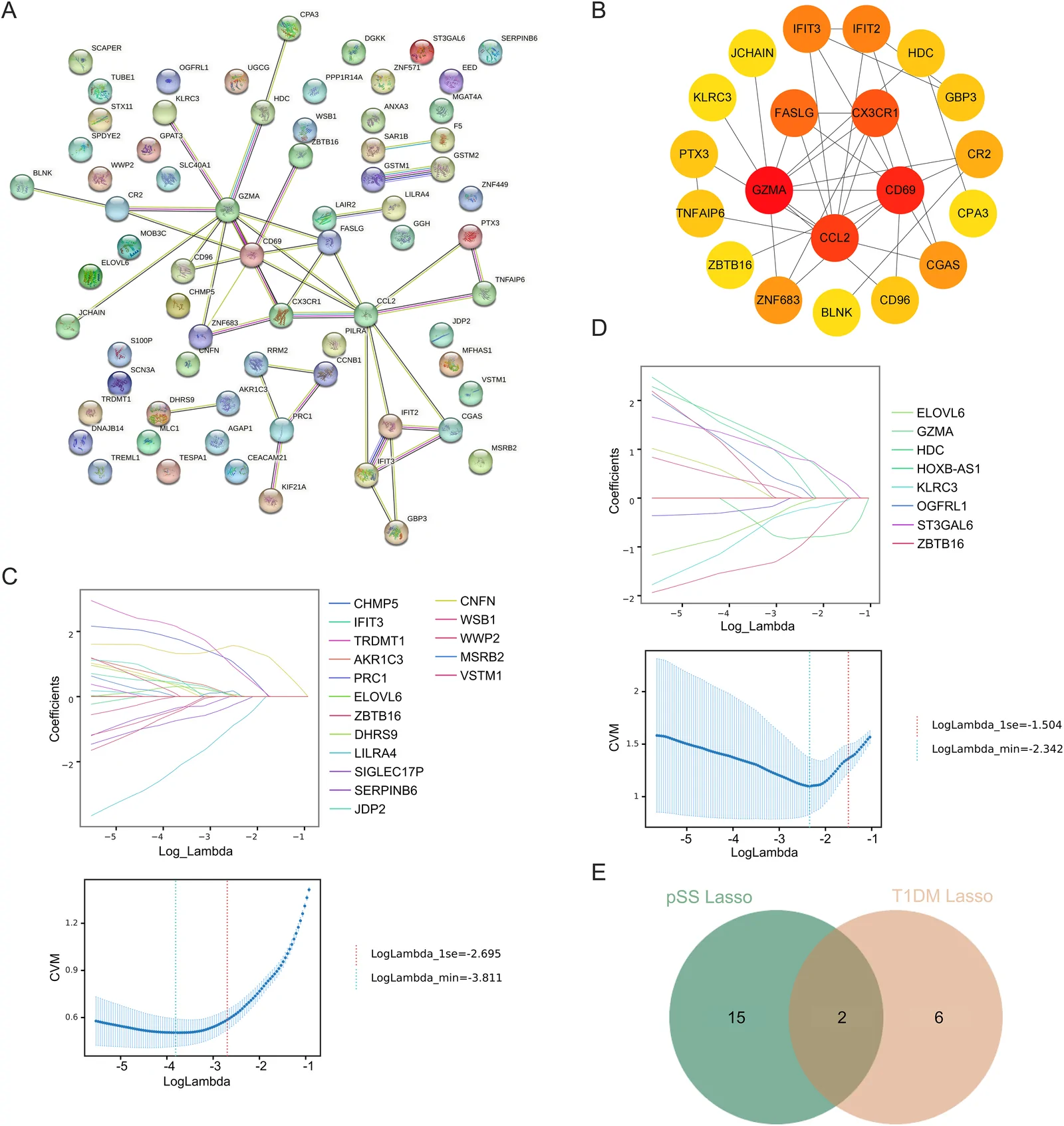
Network-based identification of shared hub genes in pSS and T1DM. **(A)** PPI network diagram of shared genes. **(B)** Cytoscape visualization of the PPI network. **(C, D)** LASSO coefficient profiles and cross-validation plots for hub-gene selection. **(E)** Venn diagram showing overlapping hub genes identified by LASSO regression in pSS and T1DM. pSS, primary Sjögren’s syndrome; T1DM, type 1 diabetes mellitus; PPI, protein−protein interaction; LASSO, least absolute shrinkage and selection operator.

To further prioritize hub candidates within the 72 shared genes, a protein–protein interaction (PPI) network was generated using the STRING database. The resulting network comprised 70 nodes connected by 42 edges, with an average node degree of 1.2, indicating a relatively sparse interaction pattern among these genes. PPI analysis identified several central genes among the 72 shared genes, including *GZMA*, *CD69*, *CCL2*, *ZNF683*, and *ZBTB16* (**Figures 4A, B**).

**Figure 5:**
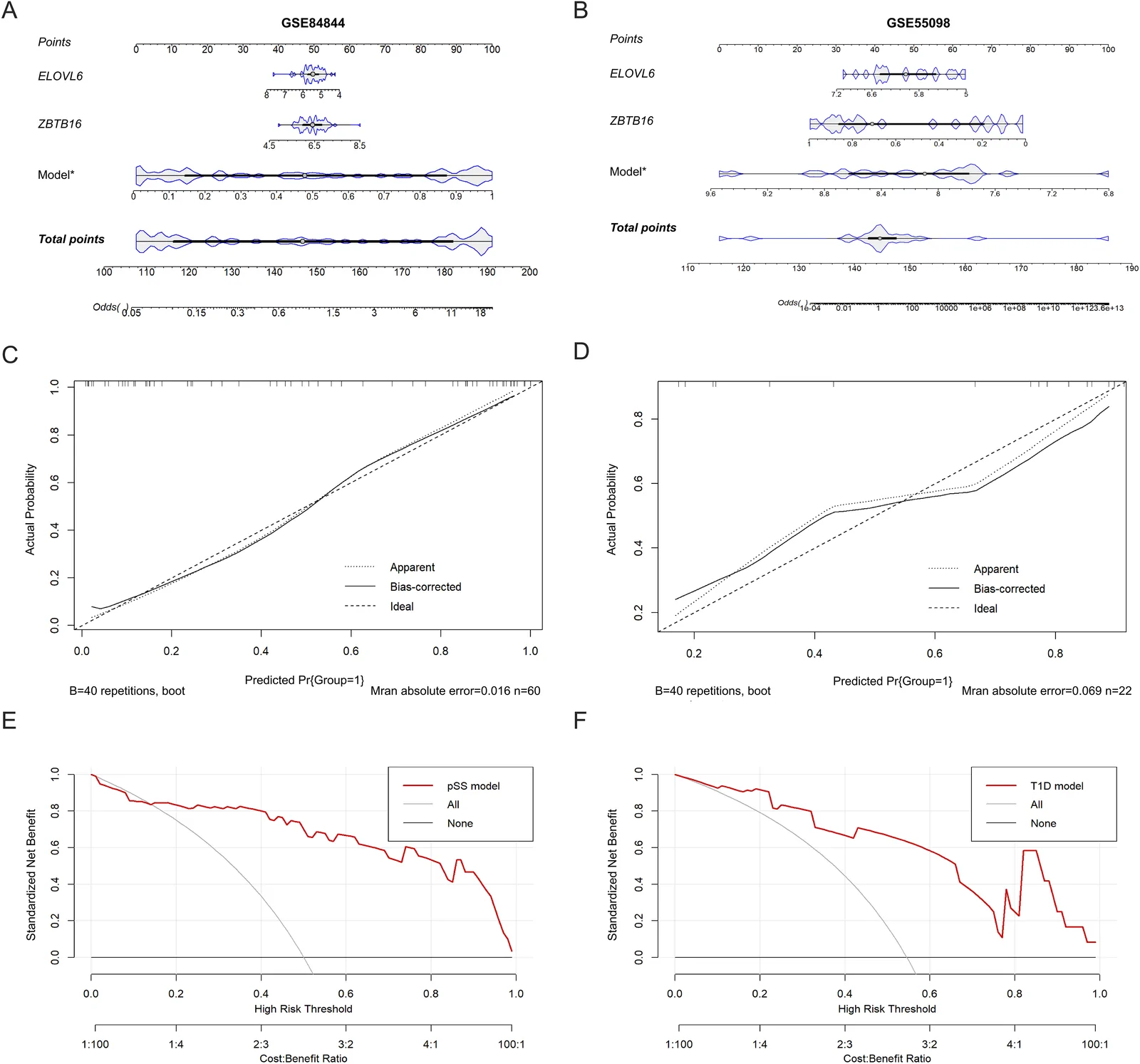
Diagnostic modeling and validation of shared signature genes. **(A, B)** Diagnostic nomograms based on two signature genes. **(C, D)** Calibration curves to assess the accuracy of the nomograms. **(E, F)** Decision curve analysis (DCA) to evaluate the net benefit of nomogram prediction of pSS and T1DM diagnostics. pSS, primary Sjögren’s syndrome; T1DM, type 1 diabetes mellitus.

To further narrow down the candidate genes LASSO regression analysis was performed using the optimal penalty parameter (λ). Cross-validation of the selected feature genes identified ELOVL6 and *ZBTB16* as the final key genes associated with both diseases. (**Figures 4C–E**). A diagnostic nomogram was subsequently developed using the screened genes via the “rms” package, demonstrating good predictive performance, which was further supported by calibration curves and decision curve analysis. (**Figures 5A–F**).

**Figure 6:**
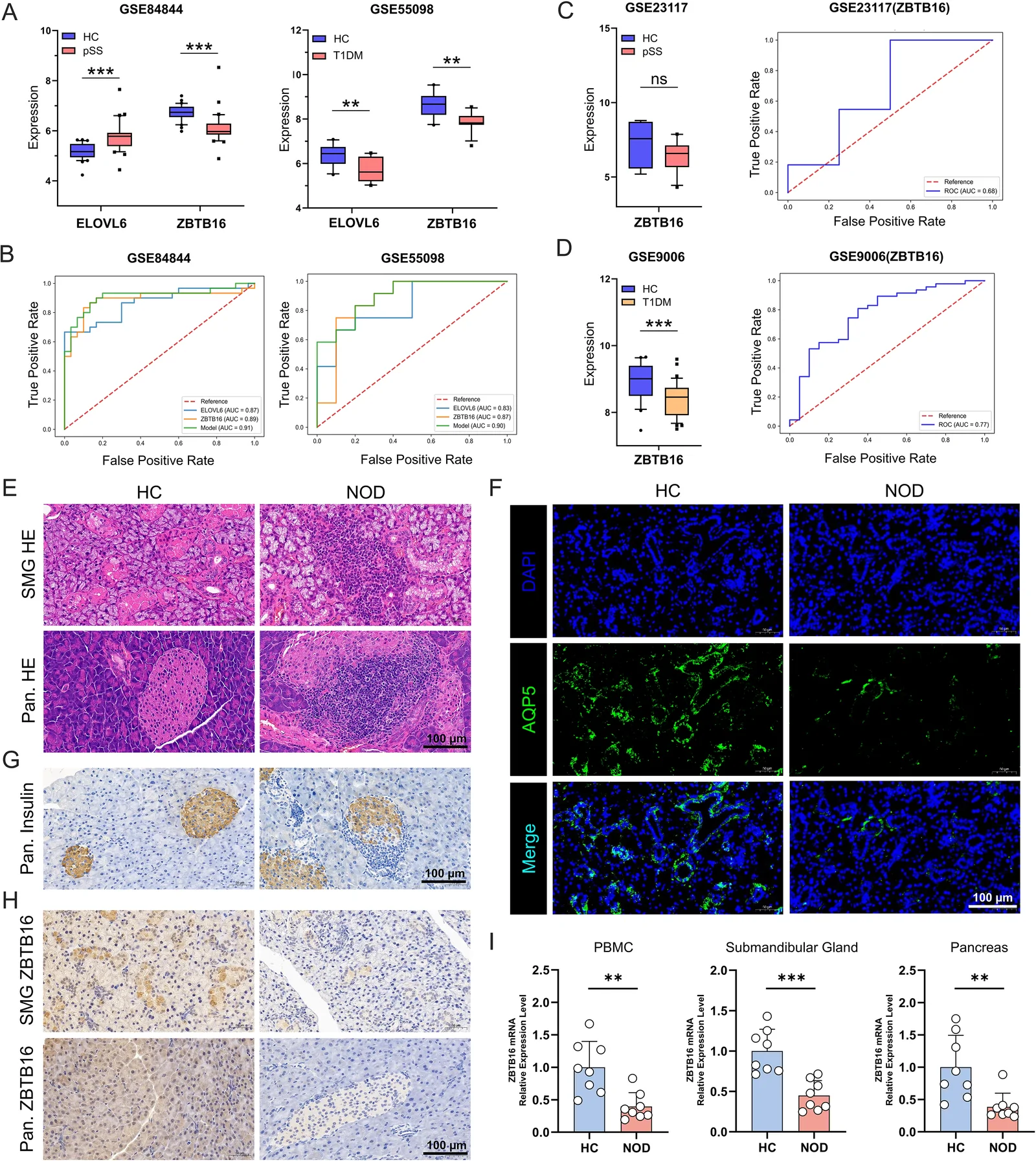
Validation of the diagnostic performance and experimental expression of *ZBTB16*. **(A)** Expression levels of *ELOVL6* and *ZBTB16* in the training cohorts. **(B)** ROC curves evaluating the diagnostic performance of *ELOVL6* and *ZBTB16*. **(C, D)** External validation of *ZBTB16* expression and diagnostic performance in GSE23117 and GSE9006. **(E)** Representative Hematoxylin and eosin (H&E) staining images of submandibular gland tissues and pancreatic tissues from control and NOD mice. **(F)** Representative immunofluorescence images of AQP5 in submandibular gland tissues. **(G)** Representative immunohistochemical staining of insulin in pancreatic tissues and *ZBTB16* in submandibular gland and pancreatic tissues. **(H)** Relative *ZBTB16* mRNA expression determined by qRT-PCR in peripheral blood, submandibular gland, and pancreatic tissues from control and NOD mice (n = 8). ROC, Receiver Operating Characteristic; SMG,. *P < 0.05, **P < 0.01, ***P < 0.001.

To further confirm the expression pattern and diagnostic value of the core hub gene in both pSS and T1DM, multi-cohort datasets were used for validation, and experimental assays were additionally performed to support the computational findings. In the training cohorts (GSE84844 and GSE55098), *ZBTB16* expression was significantly decreased in both pSS and T1DM (**Figures 6A, B**). *ZBTB16* was prioritized for downstream analysis due to its consistency across datasets. Consistent trends were observed in external validation cohorts, with statistical significance in GSE9006 (P < 0.05), while GSE23117 showed a similar but non-significant trend. Differences across datasets may reflect cohort size and platform variability. ROC analysis nevertheless supported the diagnostic potential of *ZBTB16* across datasets (**Figures 6C, D**; **Table 2**, **3**).

**Table 2:** Diagnostic test results of *ZBTB16*.

Data set	Group	Truncated value	95% CI	Sensitivity	Specificity	Youden index
GSE84844	pSS	6.33	0.79, 0.98	0.90	0.50	0.40
GSE55098	T1DM	7.97	0.70, 1.00	0.75	0.90	0.65

**Table 3:** Diagnostic test results of *ZBTB16* in external datasets.

Data set	Sample type	Platform	AUC	95% CI	Sensitivity	Specificity	Youden index
GSE23117	pSS+HC	GPL570	0.68	0.32, 1.00	1.00	0.500	0.50
GSE9006	T1DM+HC	GPL96	0.77	0.67, 0.92	0.87	0.650	0.522

To further support the bioinformatic findings, experimental validation was performed in NOD mice. H&E staining demonstrated pathological alterations in both the submandibular gland and pancreas, whereas AQP5 immunofluorescence and insulin immunohistochemistry confirmed impaired glandular function (**Figures 6E–G**). Consistent with the bioinformatic analyses, both qRT-PCR and immunohistochemistry showed significantly decreased *ZBTB16* expression in peripheral blood, submandibular gland, and pancreatic tissues from NOD mice compared with controls (**Figures 6H, I**).

### 3.3 *ZBTB16*-associated signatures implicate immune-metabolic dysregulation

**Figure 7:**
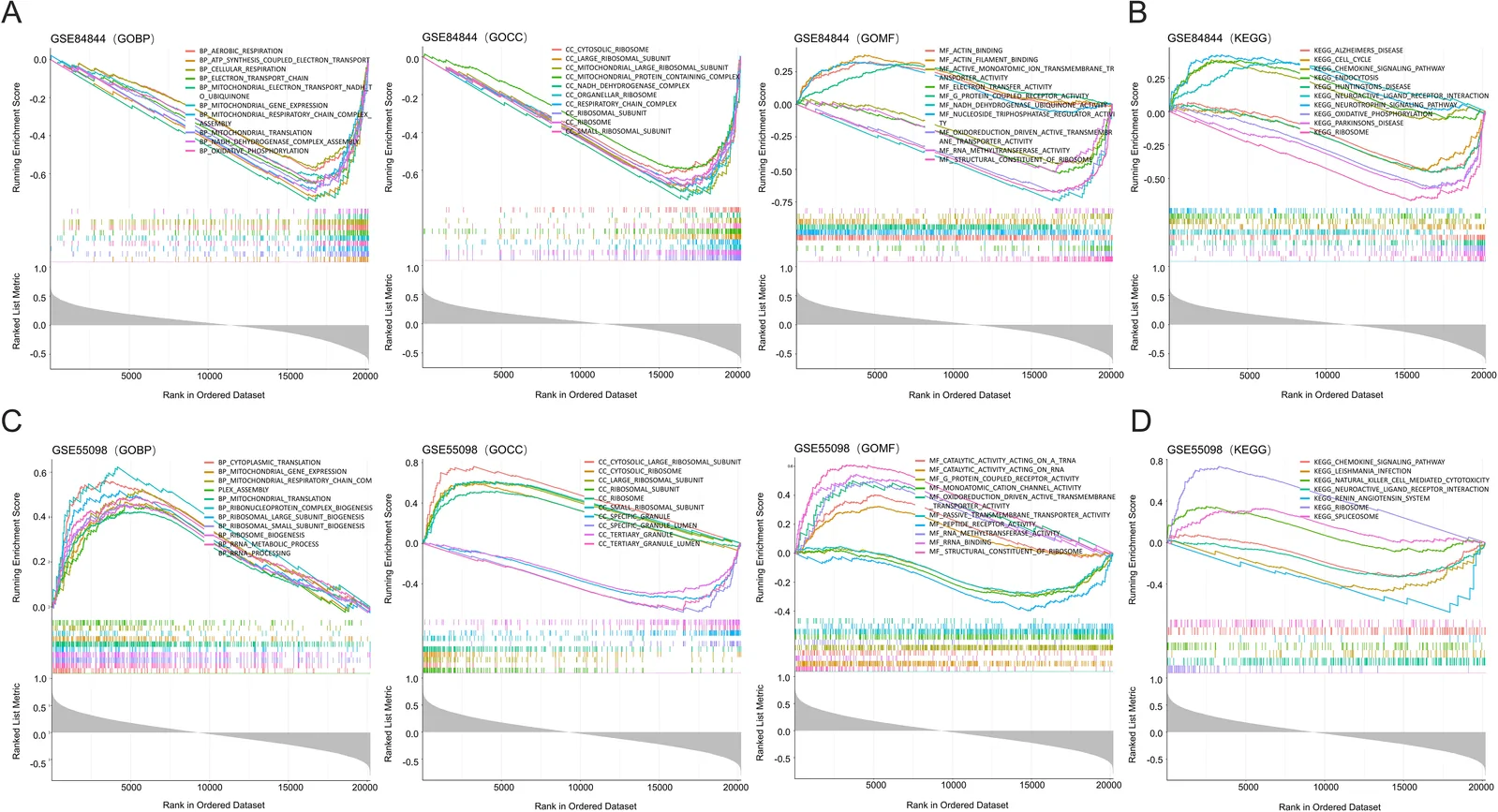
Functional characterization of *ZBTB16*-associated transcriptional signatures. **(A, C)** GO enrichment analyses, including BP, CC, and MF. **(B, D)** KEGG pathway enrichment analyses. GO, Gene Ontology; KEGG, Kyoto Encyclopedia of Genes and Genomes; BP, Biological Process; CC, Cellular Component; MF, Molecular Function.

To elucidate the biological implications of *ZBTB16*-associated transcriptional signatures, single-gene GSEA was conducted. GO enrichment analysis consistently showed that genes associated with *ZBTB16* were mainly enriched in mitochondrial activity, including energy metabolism and oxidative processes, as well as ribosome-related functions and protein synthesis. In addition, pathways related to membrane-associated signaling regulation were significantly enriched in both pSS and T1DM datasets (**Figures 7A, C**).

KEGG pathway analysis further revealed that *ZBTB16*-associated signatures were enriched in neuroactive ligand–receptor interaction and chemokine signaling pathways. Collectively, these results point to a possible role of *ZBTB16* in coordinating immune and metabolic dysregulation (**Figures 7B, D**).

### 3.4 Reduced *ZBTB16* expression is associated with decreased resting NK-cell abundance

**Figure 8:**
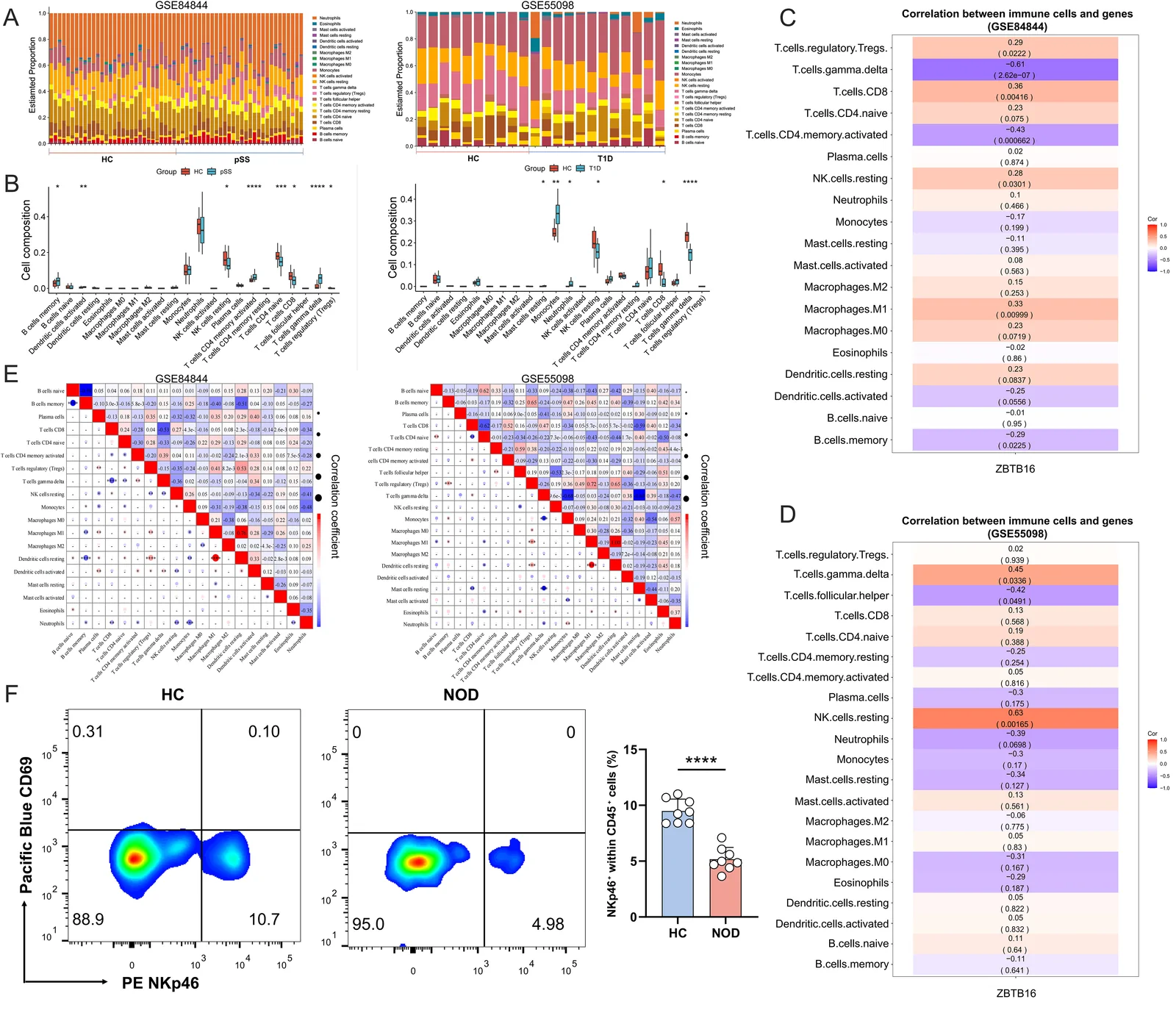
Association between *ZBTB16* expression and immune cell infiltration in pSS and T1DM. **(A)** Stacked bar plots showing the relative proportions of 22 infiltrating immune cell types estimated by CIBERSORT. **(B)** Box plots comparing immune-cell proportions between disease and control groups. **(C, D)** Spearman correlation analysis between *ZBTB16* expression and infiltrating immune cells in pSS and T1DM. **(E)** Correlation matrices showing relationships among infiltrating immune-cell populations. **(F)** Quantification of peripheral blood NK-cell proportions in control and NOD mice (n = 8). pSS, primary Sjögren’s syndrome; T1DM, type 1 diabetes mellitus. *P < 0.05, **P < 0.01, ***P < 0.001, ****P < 0.0001.

CIBERSORT analysis was performed to evaluate immune infiltration patterns shared between pSS and T1DM. The results revealed significant alterations in multiple immune cell populations. Relative to healthy control samples, resting NK cells and CD8^+^ T cells displayed consistent reductions across both autoimmune disorders. (**Figures 8A, B**). Spearman’s correlation tests uncovered a significant positive correlation between *ZBTB16* expression levels and resting NK cell proportions within both datasets (**Figures 8C, D**), whereas no consistent association was observed with the other significantly altered immune-cell subsets.

Immune cell interaction network analysis further revealed coordinated relationships among NK cells and other immune populations, including positive associations with CD8^+^ T cells in pSS and M1 macrophages in T1DM (**Figure 8D**; **Supplementary Figures S3, S4**).

To independently validate the computationally inferred reduction of NK cells, flow cytometry was performed in peripheral blood samples from HC and NOD mice. Consistent with CIBERSORT results, NK-cell proportions were significantly reduced in NOD mice compared with controls (P < 0.0001) (**Figure 8F**).

Collectively, CIBERSORT analysis and flow cytometry consistently demonstrated reduced NK-cell abundance in both datasets and the NOD mouse model. In addition, the positive correlation identified by Spearman analysis suggests that decreased *ZBTB16* expression is associated with reduced resting NK-cell abundance, although the underlying biological relationship requires further experimental investigation.

### 3.5 Exploratory identification of candidate compounds targeting *ZBTB16*

**Figure 9:**
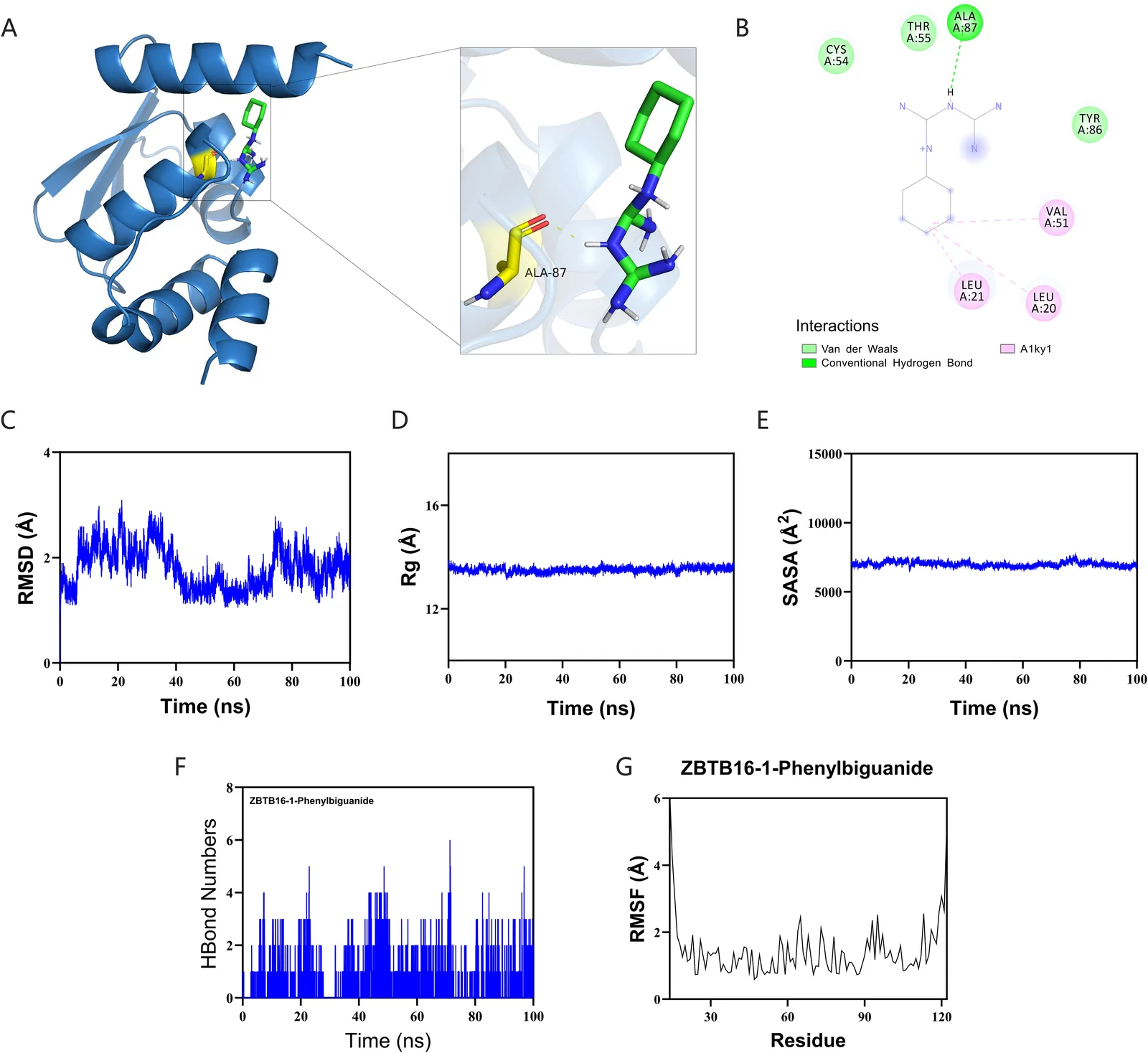
Exploratory analysis of small-molecule interaction with *ZBTB16*. **(A, B)** Molecular docking models of *ZBTB16* and 1-phenylbiguanide. **(C)** RMSD trajectory of protein-ligand complex over time. **(D)** Radius of gyration (Rg) value of protein-ligand complex over time. **(E)** Solvent-accessible surface area (SASA) value of protein-ligand complex over time. **(F)** Number of hydrogen bond (H-Bond) numbers of protein-ligand complex over time. **(G)** Root mean square fluctuation (RMSF) value of protein-ligand complex.

In an effort to uncover viable therapeutic leads, we mined the Connectivity Map (CMap) database to screen chemical compounds that could offset transcriptional alterations tied to *ZBTB16*. Among the top-ranked candidates, 1-phenylbiguanide was selected based on combined connectivity score and chemical feasibility. (**Supplementary Table 1**). Combined molecular docking and dynamic simulation data hinted at steady binding interactions occurring between *ZBTB16* and 1-phenylbiguanide (**Figures 9A–G**).

## 4 Discussion

Primary Sjögren’s syndrome (pSS) and type 1 diabetes mellitus (T1DM) are autoimmune disorders characterized by shared immune-inflammatory features, yet their conserved pathogenic mechanisms linking the two diseases remain poorly understood. In this study, integrated transcriptomic analysis identified shared immune-inflammatory and metabolic signatures between pSS and T1DM and further highlighted *ZBTB16* as a conserved hub biomarker associated with resting NK-cell alterations. These findings suggest a potential shared immunological axis linking the two disorders.

Previous work suggests that immune activation and inflammatory signaling are common features shared by pSS and T1DM (20–24). Enrichment of biological functions showed these intersecting genes primarily participate in innate immune reactions, inflammatory regulatory processes, and glutathione metabolic pathways. Glutathione is a key regulator of redox homeostasis and antioxidant defense, and its dysregulation has been associated with autoimmune-mediated tissue injury (25–27). In both pSS and T1DM, increased oxidative stress and impaired antioxidant capacity have been reported, which may contribute to glutathione depletion and redox imbalance (28). These findings suggest that altered glutathione metabolism may reflect a shared immune–metabolic disturbance contributing to sustained inflammation.

Among the shared hub genes, *ZBTB16* exhibited a consistent pattern of downregulation across multiple independent cohorts, despite minor inter-dataset variability that may reflect cohort heterogeneity and technical differences. This overall consistency, together with the results of network analysis, machine-learning algorithms, and experimental validation, supported the prioritization of *ZBTB16* as a key shared biomarker linking pSS and T1DM. Although *ELOVL6* was also identified during feature selection, it lacked consistent validation across datasets and was therefore not prioritized for further investigation. *ZBTB16* encodes a conserved transcription factor involved in immune regulation, cellular metabolism, and homeostasis (29). Several prior investigations have verified its critical function in governing immune cell differentiation and tuning mitochondrial metabolic homeostasis (30–32). Consistent with these biological functions, our GSEA analyses showed that *ZBTB16*-associated transcriptional signatures were enriched in mitochondrial activity, protein synthesis, and membrane-associated signaling pathways, suggesting that *ZBTB16* may participate in immune–metabolic regulation in both diseases.

Given the central role of mitochondrial function in redox balance, *ZBTB16*-associated metabolic alterations may be linked to oxidative stress responses and glutathione homeostasis. Redox imbalance may therefore represent a potential connection between metabolic disturbance and immune dysregulation in pSS and T1DM. Previous studies have shown that *ZBTB16* can regulate mitochondrial function and glucose metabolism in innate-like lymphocytes (29, 33, 34). In this context, reduced *ZBTB16* expression may be associated with altered immune cell function and sustained inflammatory responses, although the precise mechanisms remain to be elucidated.

Considering the role of *ZBTB16* in innate lymphocyte biology, we next examined its relationship with immune-cell infiltration patterns in both diseases. Spearman correlation analysis based on CIBERSORT-derived immune infiltration profiles consistently identified a positive association between *ZBTB16* expression and resting NK-cell abundance in both pSS and T1DM. In addition, flow cytometric analysis independently confirmed a reduction in peripheral NK-cell proportions in NOD mice, providing experimental support for the computationally predicted decrease in NK-cell abundance. However, the flow cytometric analysis was designed to validate changes in NK-cell proportions rather than the association between *ZBTB16* expression and NK cells. Therefore, the observed relationship between *ZBTB16* and resting NK cells should be interpreted as a computationally inferred association rather than a validated mechanistic interaction.

NK cells play an important role in maintaining immune homeostasis and limiting excessive immune activation (35). Reduced resting NK-cell abundance observed in both diseases may therefore reflect a disturbance in immune balance associated with autoimmune progression (36). In pSS, this may relate to glandular inflammation and lymphocytic infiltration, whereas in T1DM, it may be associated with enhanced inflammatory activation in the islet microenvironment (37–40). Since *ZBTB16* is known to regulate the biological functions of innate-like lymphocytes, its expression level is likely closely linked to the homeostatic status of NK cells. However, whether *ZBTB16* directly regulates NK-cell function requires further experimental validation. Based on these findings, *ZBTB16*-associated NK-cell alterations may represent one component of shared immune-related transcriptional alterations between pSS and T1DM. Rather than implying a defined mechanistic pathway, these results suggest coordinated changes in immune and metabolic-related gene expression programs during disease progression.

Beyond resting NK cells, CIBERSORT output revealed obvious shifts across multiple immune subsets, such as CD8^+^ T lymphocytes, regulatory T cells (Tregs), and diverse macrophage subgroups. Prior reports have confirmed the pathogenic role of CD8^+^ T cells in causing glandular damage in pSS and impairing pancreatic β-cells in T1DM (7, 11), whereas Treg dysfunction is a well-recognized mechanism underlying loss of immune tolerance in both diseases (12, 41). Macrophage polarization has also been implicated in chronic inflammatory progression and tissue remodeling (42, 43).Although these immune populations may participate in the shared pathogenesis of pSS and T1DM, the present study focused on resting NK cells because they showed the most consistent alterations across datasets and the strongest statistical association with *ZBTB16* expression. Future studies are warranted to investigate the relationship between *ZBTB16* and multiple immune cell subsets using functional and spatially resolved approaches.

As an exploratory extension, small-molecule screening identified 1-phenylbiguanide as a candidate compound with potential interaction with *ZBTB16*. Simulations of molecular docking and dynamics demonstrated robust and steady interaction between the targeted compound and *ZBTB16*. Even so, all these outcomes stem purely from computational modeling, lacking experimental proof regarding bioactivity, target binding and therapeutic effectiveness. Accordingly, any potential link between 1-phenylbiguanide and pSS or T1DM must be viewed cautiously. Follow-up cellular and animal experiments are necessary to clarify whether this predicted binding carries biological functionality.

Several limitations should be acknowledged. First, this study establishes associations rather than direct causality. Second, immune infiltration was inferred computationally and requires experimental confirmation. Third, discovery of 1-phenylbiguanide depended entirely on computational modeling pipelines, with no supporting experimental data to confirm its binding properties and biological impacts. Its prospective clinical utility thus cannot be reliably established. Nevertheless, by integrating bioinformatics analyses with experimental validation, this study provides evidence supporting *ZBTB16* as a shared biomarker and suggests a potential *ZBTB16*-associated immune-metabolic axis in pSS and T1DM.

## 5 Conclusion

In summary, this study identified shared immune-inflammatory and metabolic signatures between pSS and T1DM and highlighted *ZBTB16* as a conserved hub biomarker across the two autoimmune disorders. Integrated bioinformatics analyses combined with experimental validation suggested a potential *ZBTB16*-associated immune-metabolic crosstalk involving resting NK-cell alterations, suggesting a potential shared immunological axis linking pSS and T1DM.These findings support *ZBTB16* as a potential diagnostic biomarker and a candidate therapeutic target for both diseases.

Nevertheless, the present study establishes associations rather than causality. The precise mechanisms by which *ZBTB16* may regulate immune-metabolic imbalance and NK-cell homeostasis remain to be clarified. Future studies using functional experiments and NOD mouse models are warranted to validate the mechanistic role of *ZBTB16* in regulating NK-cell homeostasis in shared autoimmune pathogenesis.

## Data Availability

The original contributions presented in the study are included in the article/**Supplementary Material**. Further inquiries can be directed to the corresponding author.
