# Supplementary material for: ZBTB16-associated NK cell alterations reveal shared immunometabolic signatures linking primary Sjögren’s syndrome and type 1 diabetes mellitus
Source: Front Immunol. 2026 Aug 18;17:1871048. doi: 10.3389/fimmu.2026.1871048 (PMC13529952; doi:10.3389/fimmu.2026.1871048)
Supplement: Supplementary file 1 [file DataSheet1.docx]

**Supplementary Material**

Supplementary Figure 1 GSE84844WGCNA Analysis. (A) Sample clustering diagram; (B) Relationship between the fitting parameters of the scale-free topological model and the soft threshold; (C) Relationship between the average connectivity and the soft threshold; (D) Construction of gene co-expression modules; (E) Clustering tree among modules and clustering heatmap among modules; (F) Heatmap of the relationship between genes in each module and the module.

Supplementary Figure 2 GSE55098WGCNA Analysis. (A) Sample clustering diagram; (B) Relationship between the fitting parameters of the scale-free topological model and the soft threshold; (C) Relationship between the average connectivity and the soft threshold; (D) Construction of gene co-expression modules; (E) Clustering tree among modules and clustering heatmap among modules; (F) Heatmap of the relationship between genes in each module and the module.

Supplementary Figure 3 A heatmap showing the correlations between different immune cells in pSS and different groups. Red represents positive correlation, white represents no correlation, and blue represents negative correlation.

Supplementary Figure 4 A heatmap showing the correlations between different immune cells in T1D and different groups. Red represents positive correlation, white represents no correlation, and blue represents negative correlation

Supplementary Figure 5 Flow cytometric gating workflow to distinguish NK cells. (A) Lymphocytes were isolated using FSC-A versus SSC-A dot plots. (B) CD45-positive immune cells were sorted from the lymphocyte gate by APC-CD45 staining. (C) NK cells were characterized as PE-NKp46⁺ cells among CD45⁺ lymphocytes. Pacific Blue-CD69 expression was applied to separate resting (CD69⁻) and activated (CD69⁺) NK cell subsets.

Supplementary Table 1 Screening results of small molecule drugs (Top 5)

| Rank | Score | Type | ID | Name | Description |
| --- | --- | --- | --- | --- | --- |
| 8539 | -96.81 | cp | BRD-K67445247 | flurofamide | bacterial urease inhibitor |
| 8529 | -91.9 | cp | BRD-A41722204 | sulmazole | calcium sensitizer, phosphodiesterase inhibitor |
| 8520 | -91.33 | cp | BRD-K31491153 | 1-phenylbiguanide | serotonin receptor agonist |
| 8517 | -91.29 | cp | BRD-K59773493 | benzohydroxamic-acid | metal chelator, antibacterial |
| 8518 | -91.06 | cp | BRD-K21009077 | BRD-K21009077 | dual specificity protein phosphatase inhibitors |

Supplementary Table 2 The primer sequence

| Gene | Forward sequence 5'-3' | Reverse sequence 5'-3' |
| --- | --- | --- |
| GAPDH | CCTCGTCCCGTAGACAAAATG | TGAGGTCAATGAAGGGGTCGT |
| ZBTB16 | ACATACGGGTGTGAACTCTGCG | TGAGAACTGGGCACCGCATTGA |
